# Metabolic profile in women with bulimia nervosa or binge-eating disorder before and after treatment: secondary analysis from the randomized PED-t trial

**DOI:** 10.1007/s40519-023-01567-y

**Published:** 2023-04-27

**Authors:** Therese Fostervold Mathisen, Jorunn Sundgot-Borgen, Jan H. Rosenvinge, Solfrid Bratland-Sanda, Mette Svendsen, Gunn Pettersen, KariAnne Vrabel, Oddgeir Friborg

**Affiliations:** 1 Faculty of Health, Welfare and Organisation, Østfold University College, Fredrikstad, Norway; 2 Institute of Sports Medicine, Norwegian School of Sport Sciences, Oslo, Norway; 3 Department of Psychology, Faculty of Health Sciences, UIT, The Arctic University of Norway, Tromsø, Norway; 4 Department of Outdoor Studies, Sports and Physical Education, University of South-Eastern Norway, Bø, Norway; 5 Department of Endocrinology, Obesity and Preventive Medicine, Oslo University Hospital and Department of Nutrition, Institute of Basic Medical Sciences, University of Oslo, Oslo, Norway; 6 Department of Health and Caring Sciences, Faculty of Health Sciences, UIT, The Arctic University of Norway, Tromsø, Norway; 7 Department of Psychology, Research Institute of Modum Bad, Vikersund, Norway

**Keywords:** Bulimia nervosa, Binge-eating disorder, Cholesterol, Blood glucose, Thyroid hormone, Physical health

## Abstract

**Purpose:**

Chaotic eating and purging behavior pose a risk to the metabolic health of women with bulimia nervosa (BN) and binge-eating disorder (BED). This study reports on one-year changes in blood markers of metabolic health and thyroid hormones in women with BN or BED attending two different treatments.

**Methods:**

These are secondary analyses from a randomized controlled trial of 16-week group treatment of either physical exercise and dietary therapy (PED-t) or cognitive behavior therapy (CBT). Blood samples collected at pre-treatment, week eight, post-treatment, and at 6- and 12-month follow-ups were analyzed for glucose, lipids (triglycerides (TG), total cholesterol (TC), LDL cholesterol (LDL-c), HDL cholesterol (HDL-c), apolipoprotein A (ApoA) and apolipoprotein B (ApoB) lipoproteins), and thyroid hormones (thyroxine (T4), thyroid stimulating hormone (TSH), and thyroperoxidase antibodies).

**Result:**

The average levels of blood glucose, lipids and thyroid hormones were within the recommended range, but clinical levels of TC and LDL-c were detected in 32.5% and 39.1%, respectively. More women with BED compared with BN had low HDL-c, and a larger increase over time in TC and TSH. No significant differences occurred between PED-t and CBT at any measurement. Exploratory moderator analyses indicated a more unfavorable metabolic response at follow-up among treatment non-responders.

**Conclusion:**

The proportion of women with impaired lipid profiles and unfavorable lipid changes, suggests active monitoring with necessary management of the metabolic health of women with BN or BED, as recommended by metabolic health guidelines.

*Level of evidence:* Level I: Evidence obtained from a randomized, experimental trial.

*Trial registration number:* This trial was prospectively registered in the Norwegian Regional Committee for Medical and Health Research Ethics on December 16, 2013, with the identifier number 2013/1871, and in Clinical Trials on February 17, 2014, with the identifier number NCT02079935.

## Background

In a psychotherapy setting, there may be limited attention to safeguard the somatic health of persons suffering from bulimia nervosa (BN) or binge eating disorder (BED). Weight fluctuations and dysfunctional eating patterns due to recurrent binge eating episodes and purging methods like fasting, vomiting, misuse of laxatives, and excessive physical exercise [1, 2], may increase the risk of hyperlipidemia [3]. Additionally, large variability in the distribution of daily energy intake may impair cholesterol regulation [4], and persistent variations in blood glucose levels increase the risk of both type 2 diabetes mellitus [5] and cardiovascular diseases [6]. These factors may be reasonable explanations for the increased risk of metabolic disorders among patients having these ED diagnoses [7–13]. Corroborating these findings, a large longitudinal cohort study (*N* = 416,709) reported the risk of any cardiovascular diseases as four times higher among BN as compared to control cases [14]. Considering the frequent binge-eating episodes in BED and no compensatory behavior, it is reasonable to expect worse plasma profiles of glucose and lipids in persons with BED compared to those with BN. Previous cross-sectional studies have reported plasma lipid profiles in BN and BED of clinical concern, but little is known about the ability of persons with BN or BED to achieve long-term improvements in their lipid- and glucose regulation following treatment [15]. One may expect that a lower frequency of binges and purging may improve metabolic control.

Knowledge from the field of physiology and weight regulation points towards a metabolic adaption with increasing weight loss, or also due to eating restriction and/or extreme weight loss practices irrespective of weight reduction per se [16, 17]. This may explain previous findings of reduced resting metabolic rate in as much as every third patient with BN or BED [18–20]; yet with a poor understanding of the pathophysiology for their metabolic suppression [19, 21]. The finding that individuals with BED at age 16 already at the age of seven had a significantly higher BMI than a reference group, is of notice [22], and suggests a disposition for disruptions in body weight regulation. These findings may indicate two potential mechanisms for reduced metabolism in persons with BN or BED: (a) one that is acquired through repetitive periods with irregular or restrictive eating practice, and that may be responsive to successful ED treatments, and (b) hormonal alterations due to undetected clinical or subclinical hypothyroidism that can exacerbate the development of BN or BED, and which is not corrected during the ED treatment. Resting metabolic rate has been reported to normalize during the treatment of BN in malnourished inpatients [20]. Still, we lack knowledge of the prevalence of hypothyroidism in individuals with BN or BED, specifically for the overweight or obese BMI range, as well as how thyroid hormones respond to ED treatment within these diagnostic groups.

Some methodological issues with the current studies of metabolic and thyroid markers among persons with BN and BED may be noted: the practice of blood sampling in post-prandial conditions, deficient reporting on the analyzed cholesterol- or plasma lipid fractions, lack of comparisons with recommended levels as devised by clinical guidelines, as well as the lack of prospective study designs [7–13, 22]. Our research group has reported negligible treatment effects among women with BN or BED regarding body weight, body composition and fitness criteria indicative of risk profiles associated with non-communicable diseases following cognitive behavior therapy (CBT) or physical exercise- and dietary therapy (PED-t) [23]. Despite the lack of noticeable somatic health improvements [23], we did not consider if the metabolic profiles (i.e., levels of plasma lipids and glucose) or the thyroid status (i.e., TSH, thyroid stimulating hormone; T4, thyroxine; or anti-thyroperoxidase, TPO) may have improved following participation in any of the two intervention arms.

Physical activity can improve glycemic control and plasma lipid levels by increased muscle metabolism and by the production of signal substances (myokines) with metabolic effects in organ tissues like fat tissue and the liver [24, 25]. Recent findings highlight the beneficial effect of regular physical activity on total mortality irrespective of body weight status [26]. In our treatment trial, we found no differences in cardiorespiratory fitness and accelerometer-assessed physical activity between the intervention groups and observed no changes by time within the groups [27]. However, the PED-t intervention caused significantly increased maximal muscle strength and lean body mass compared to CBT [23]. There is a well-known limitation that accelerometers underestimate resistance-related exercise and physical activity [28] which makes it reasonable to suggest that the participants in PED-t performed more *resistance* exercise than participants in CBT. The importance of resistance training and gaining muscle mass justify expectations of participants in PED-t to improve their metabolic health (i.e., blood lipids and glucose) more than participants in CBT.

The current study contributes to the accumulation of knowledge on how remission, or regular physical activity, may affect metabolic health in individuals with BN or BED; hence, the aims of this study were:

1. To describe the pre-treatment metabolic profile (i.e., blood lipids and glucose) and thyroid hormones of women with BN or BED.
2. To examine immediate and one-year changes in the metabolic profile and thyroid hormones following treatment with either PED-t or CBT.
3. To explore the role of remission in effecting metabolic and thyroid hormonal changes.

We hypothesized that (1) at pre-treatment women with BN or BED will display an impaired metabolic profile, and more notably among those presenting with BED; (2) that responding to either CBT or PED-t will improve metabolic profile, but that (3) the PED-t will surpass the CBT in improving metabolic profile because of its focus on regular eating and physical activity.

## Methods

### Study procedures and design

This paper presents secondary outcome analyses from a randomized controlled trial (RCT) evaluating the effect of two treatment interventions for BN and BED (CBT versus PED-t versus waitlist references) [29, 30]. Further details on the recruitment, power calculations, randomization, and therapy content are provided elsewhere [29, 30]. Briefly described, the CBT was arranged according to Fairburn’s individual CBT-E therapy but adapted for groups. The PED-t consisted of one weekly group meeting with supervised, progressive resistance exercise directly followed by dietary therapy, and homework for two additional exercise sessions (one resistance exercise- and one interval running session). As waitlist participants did not donate blood samples, the waitlist participants are not included herein.

Fasting blood samples (> 8 h fast) were extracted five times from persons actively participating in either treatment, i.e., at pre-treatment, mid-treatment (week eight), post-treatment (week 17) and at follow-up (week 26 and 52, respectively); hence, in the present study the design was mixed factorial with *Group* (CBT vs. PED-t) and *Time* (repeated measures) as between and within factors, respectively.

### Participants

**Fig. 1:**
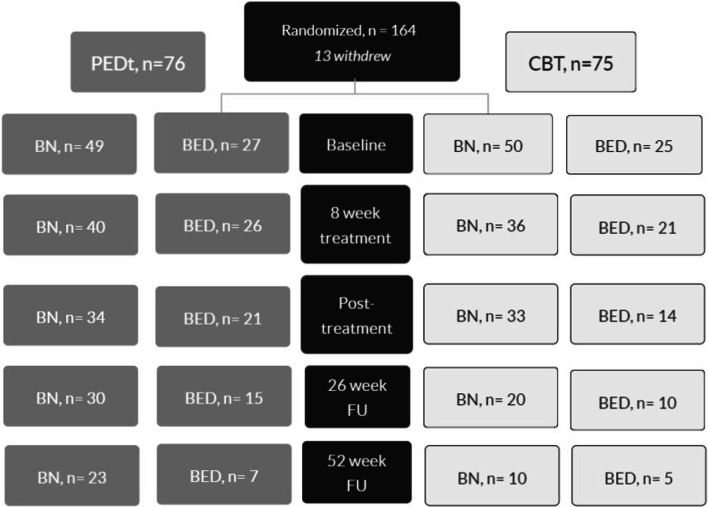
Flow of participants providing blood samples throughout the study. *BN* bulimia nervosa, *BED* binge-eating disorder, *PED-t* physical exercise and dietary therapy, *CBT* cognitive behavior therapy, *FU* follow-up

Participants (n = 151) were women with BN or BED, aged 18–40 years and with BMI 17.5–35 kg/m^2^, recruited for outpatient treatment with either 20 weekly group treatment sessions of CBT or PED-t in 2014–2016. All participants signed an informed consent before participation in the original trial. The number of recruited and retained participants which met physically in our lab and gave blood samples, is illustrated in Fig. 1.

### Outcomes

Taken from blood samples the outcome variables were lipids (total cholesterol (TC), low density lipoprotein cholesterol (LDL-c), high density lipoprotein cholesterol (HDL-c), apolipoprotein A (ApoA), apolipoprotein B (ApoB), triglycerides (TG)), glucose, and thyroid hormones (thyroxine (T4), thyroid stimulating hormone (TSH), and thyroperoxidase antibodies (anti-TPO)). These variables were also presented categorially as the frequency of participants with values above clinical recommendations pre-treatment.

Visceral adipose tissue (VAT) was measured on overnight fasted subjects between 07.30–11.00AM by dual-energy X-ray absorptiometry (Lunar iDXA, version 14.10.022 GE Healthcare, Madison, WI, USA), and analyzed by a trained technician. The machine was calibrated daily, and the measurement was by a three-site scan (including the enCORE software program for measurement and calculation of VAT).

In the current paper, the status being in *remission* embraces those in full- and partial remission (responding to treatment), following the definitions outlined in our previous paper [29]. Here, “full remission” was defined as no binge- or purging behavior the past 28 days and a national, normative global mean score (including 1 standard deviation) on the Eating Disorder Examination questionnaire below cut-off (EDE-q) ≤ 2.35 [29, 31]. Moreover, “partial remission” was defined as < 4 binge- or purging episodes the past 28 days, and a global score of EDE-q below national, clinical cut-off ≤ 2.6 [29]. As previously reported [29], among those receiving PED-t in total 48.9% was in post-treatment remission, 37% at week 26, and 39.3% at week 52. The corresponding results for CBT were 28.7%, 24.7%, and 43.7%, respectively.

#### Blood samples and analyses

Blood sampling was performed by lab assistants, filling two 8.5 ml serum tubes with a gel layer per participant. Each tube was left for 20 min before being centrifuged with 3400 RPM (2000 G) for 10 min, and finally transferred to 5-ml Sarstedt tubes and stored frozen (-80˚C). All samples were analyzed by a professional lab (Fürst, Oslo, Norway) according to standard procedures.

#### Glucose

Glucose levels of 4 – 5.9 mmol/L were considered normal, with levels ≤ 3.9 mmol/L considered low, and levels ≥ 6 mmol /L as high blood glucose.

#### Lipids

Lipid analyses were performed for TG, TC, LDL-c, HDL-c ApoA and ApoB. Guidelines and clinical cut-offs were applied for (here: values indicate the recommended levels) TG (< 1.7 mmol/L), TC (≤ 5 mmol/L), LDL-c (≤ 3 mmol/L), HDL-c (≥ 1.2 mmol/L), ApoA (> 1.4 g/L), ApoB (< 1 g/L), and ApoB/ApoA ratio (< 1) [32].

#### Thyroid hormones

Normal TSH, T4 and anti-TPO levels were considered to be 0.5 – 3.6 mIU/L, 11.0 – 23.0 pmol/L, and < 100kU/L, respectively [33, 34]. The following cut-offs were applied for evaluation of deviations from normality [33] with respect to hyperthyroidism (if TSH < 0.2 mIU/L), primary hypothyroidism (if TSH > 3.6 mIU/L and T4 < 10 pmol/L), and subclinical hypothyroidism (if TSH > 3.6 and T4 > 10 pmol/L)[34]. Signs of autoimmune condition were considered present if anti-TPO > 100 kU/L. A total of 9 women (8 with BN) had extreme and exact similar levels of anti-TPO (1300 kU/L) throughout the period. These are included in the baseline characterization, but as we evaluated these as extreme outliers, we chose to eliminate them from the analyses of this variable.

### Statistical analyses

The IBM SPSS 29 was used for all analyses. Summary statistics are presented for demographic and pre-treatment characteristics of the participants in terms of means/medians or frequencies. Distributional form and dispersion are described in terms of skewness and kurtosis (Z-values) and standard deviations (SD), respectively. Pre-treatment differences between BN and BED were examined with independent t-test or Mann–Whitney tests. The latter was used if skewness or kurtosis surpassed a Z-values of 4. Mean scores differences were quantified as Hedge’s *g* effect sizes and interpretations were based on the classifications of Cohen [35] where ≤ 0.2, 0.2–0.5 and > 0.8 represented a small, medium and strong effect size, respectively.

Generalized linear mixed models (GLMM) were fit to examine intervention effects and moderator variables as effect-modifiers. Dependencies between the repeated observations were accounted for by estimating a compound symmetry or first-order auto-regressive coefficients to account for correlations in the residual matrix depending on which model fit best according to the Bayesian Information Criterion (preferring the model with the lowest BIC value). The standard errors were estimated using the restricted maximum likelihood procedure and adjusted with the robust sandwich estimator due to departures from a normal distribution (especially in terms of kurtosis) for some of the variables (Table 1). Degrees of freedom were calculated using the Satterthwaite approximation, and type III F-tests were used to assess the statistical significance of the fixed factors. Due to multiple testing, null-hypotheses were dismissed at an alpha level < 0.01 with a corresponding 99% confidence interval.

**Table 1:** Baseline demographics, lipid and metabolic parameters per diagnosis

	Clinical level*	Skew / Kurt *Z*-values	BN (*n* = 99) *M* / *Md* (*SD*)	BED (*n* = 52) *M* / *Md* (*SD*)	*g*	*p*
General characteristic						
Age (yrs)		1.44/− 1.75	28.1/28.0 (5.4)	29.6/29.7 (6.1)	− 0.28	.11
BMI (kg/m^2^)		3.38/− 0.33	23.5/23.2 (3.6)	29.2/28.5 (5.0)	− 1.38	< .001
AN-history (*n*, %)			26 (25.5%)	5 (2.6%)		.004
EDE-q global	> 2.6		3.76/3.85 (0.9)	3.53/3.72 (0.9)	0.26	0.35
Binge-eating (no.)	< 4		12.1/10.0 (10.2)	11.48/10.0 (11.7)	0.05	0.65
Purging (no.)	< 4		28.35/20.0 (33.2)	9.1/5.0 (10.8)	0.70	< .001
Glucose (mmol/L)	4.0–5.9	7.33/13.93	4.49/4.43 (0.42)	4.92/4.88 (0.62)	− 0.86	< .001
Lipoproteins						
Total cholesterol (mmol/L)	< 5.0	2.54/1.79	4.67/4.60 (0.93)	4.72/4.70 (0.73)	− 0.07	.70
LDL (mmol/L)	< 3.0	3.16/0.74	2.84/2.76 (0.92)	3.02/2.90 (0.81)	− 0.21	.24
HDL (mmol/L)	> 1.2	4.94/4.11	1.67/1.57 (0.41)	1.59/1.48 (0.45)	0.20	.14
Triglycerides (mmol/L)	< 1.7	8.88/11.76	0.96/0.86 (0.46)	1.10/1.00 (0.49)	− 0.29	.05
ApoB (g/L)	< 1.0	2.64/0.34	0.79/0.78 (0.20)	0.83/0.77 (0.18)	− 0.20	.25
ApoA (g/L)	> 1.4	4.35/3.08	1.42/1.40 (0.23)	1.42/1.38 (0.26)	0.02	.58
Lipid ratios						
Total cholesterol/HDL	< 5.0	3.64/0.43	2.89/2.78 (0.66)	3.15/3.05 (0.79)	− 0.37	.03
ApoB/ApoA	< 1.0	2.42/0.39	0.56/0.54 (0.14)	0.59/0.57 (0.16)	− 0.25	.16
Thyroid parameters						
TSH (mU/L)	0.5–3.6	7.72/10.59	2.05/1.86 (0.85)	2.31/2.11 (1.08)	− 0.28	.23
T4 (pmol/L)	11.0–23.0	0.79/-0.99	14.55/14.36 (1.92)	14.79/14.70 (1.78)	− 0.13	.46
Anti-TPO (kU/L)	< 100	18.87/31.93	150.81/43.35 (348.88)	61.93/41.5 (95.3)	0.31	.39
Anti-TPO (kU/L) ^1^	< 100	44.91/232.02	46.34/42.10 (20.86)	61.93/41.5 (95.3)	0.26	.97

*Skew*-skewness, *Kurt* kurtosis, *M* mean, *Md* median, *SD* standard deviation, g-Hedge’s *g*-standardized mean difference, *p*-probability for H0 (*F*- or Mann–Whitney *U* tests). *BMI* body mass index, *AN* Anorexia Nervosa, *HDL* high density lipoprotein, *LDL* low density lipoprotein, *ApoB* apolipoprotein B, *ApoA* apolipoprotein A, *TSH* thyroid stimulating hormone, *T4* thyroxine, *Anti-TPO* anti-thyroid peroxidase, Purging, total episodes with self-induced vomiting, use of laxative, or driven exercise, *Recommended clinical levels[1–4], (^1^Nine cases with extreme scores (= 1300) were removed, all being BN cases)

The base model included *Group* (CBT vs PED-t), *Time* (outcome repeatedly measured) as fixed factors and the interaction term Group*Time. The baseline score for the outcome was added to all models in order to adjust for an imperfect randomization and the substantial correlations between the baseline and the follow-up measures, which increases the statistical power [36]. We also included pre-treatment ED *diagnosis* (BN or BED), *remission* status (ED diagnosis maintained or lost), degree of *purging* behavior (as z-score) and baseline visceral adipose tissue (VAT in kilograms) as covariates. These were first examined as simple correlations with the outcome (crude model), and only significant covariates were retained in the final adjusted model. Since *Remission* and *purging* were repeatedly assessed they were treated as time-variant covariates. Finally, we explored if baseline ED *diagnosis* and post-intervention *Remission* status moderated the outcome by adding them as two- and three-way effect-modifiers by *Group* and *Time*.

## Results

Pre-treatment characteristics as well as metabolic and thyroid hormonal levels in the BN and BED diagnostic groups are presented in Table 1. Other than the number of purging episodes, only BMI and glucose were significantly different between the ED diagnoses (*p* < 0.001).

**Fig. 2:**
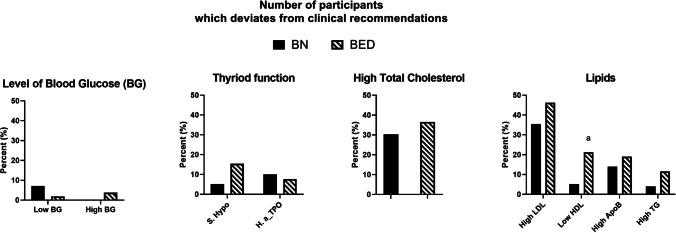
Percentages below and above defined clinical metabolic profile cut-offs at pre-treatment. *BN* bulimia nervosa; *BED* binge-eating disorder, *S.Hypo*,= subclinical hypothyroidism; *H.a_TPO* high anti-TPO, *TG* triglycerides, *LDL* low density lipoprotein, *HDL* high density lipoprotein, *ApoB* apolipoprotein B; ^a^significant difference, *p* < 0.001

Figure 2 displays pre-treatment numbers of participants with glucose levels below and above recommendations, and lipid levels above recommendations. Notably, in total 32.5% and 39.1% of the participants showed above recommended levels in TC and LDL-c, respectively. No differences were found between women with BN or BED on these categorical evaluations pre-treatment except that low HDL-c was more frequent in women with BED (21.2%) compared to BN (5.1%) (proportion difference = 16.1%, 99% CI = 0.3–32.4%, *p* = 0.002).

### Change in lipid and hormone markers depending on pre-treatment BN or BED diagnosis

ED diagnosis was not significant as a treatment effect-modifier in any of the outcome analyses. The prospective development in the outcome measures for the BN and BED diagnoses is presented in Table 2. Compared to women with a pre-treatment diagnosis of BN, women with a diagnosis of BED had significantly less favorable lipid and metabolic levels than women with a diagnosis of BN. Contrast analyses showed increasing levels of TC and TSH in BED subjects whereas BN subjects remained stable, which was significantly different at the final follow-up (*p* < 0.001 and *p* = 0.003, respectively). Other lipid measures showed a similar but less reliable difference at the final follow-up for LDL-c (*p* = 0.02), and a generally higher level of T4 across the entire follow-up period among BED than among BN subjects (*p*’s = 0.01, 0.04, 0.02 and 0.04).

**Table 2:** Development of lipoprotein and metabolic levels depending on eating disorder diagnosis (based on final adjusted model)

	Estimated marginal mean _99% confidence interval_			
	Mid 8 week	Post 17 week	26 week	52 week
Total cholesterol (mmol/L)				
BN	na	4.55 _4.39–4.72_	4.63 _4.40–4.85_	4.42 _4.14–4.70_
BED		4.55 _4.25–4.85_	4.91 _4.61–5.21_	5.02 _4.71–5.32_
*M* diff (*p*)		0 (.97)	− 0.28 (.04)	**− 0.60 (< .001)**
LDL (mmol/L)				
BN	na	2.79 _2.64–2.93_	2.86 _2.64–3.08_	2.79 _2.56–3.01_
BED		2.81 _2.55–3.08_	3.14 _2.83–3.44_	3.16 _2.81–3.51_
*M* diff (*p*)		− 0.02 (.82)	− 0.28 (.06)	− 0.37 (.02)
HDL (mmol/L)				
BN	1.65 _1.58–1.72_	1.58 _1.51–1.65_	1.59 _1.5–1.67_	1.50 _1.39–1.62_
BED	1.62 _1.52–1.73_	1.53 _1.44–1.63_	1.61 _1.5–1.73_	1.62 _1.48–1.77_
*M* diff (*p*)	0.03 (.61)	0.05 (.27)	− 0.02 (.64)	− 0.12 (.10)
Triglycerides (mmol/L)				
BN	1.00 _0.91–1.09_	0.98 _0.88–1.08_	1.03 _0.91–1.14_	0.95 _0.79–1.10_
BED	1.08 _0.91–1.25_	0.99 _0.82–1.16_	1.24 _0.94–1.54_	1.26 _0.58–1.94_
*M* diff (*p*)	− 0.08 (.31)	− 0.01 (.95)	− 0.21 (.09)	− 0.31 (.15)
ApoB (g/L)				
BN	0.80 _0.77–0.83_	0.78 _0.75–0.81_	0.81 _0.77–0.86_	0.79 _0.75–0.84_
BED	0.81 _0.78–0.85_	0.77 _0.72–0.82_	0.86 _0.79–0.94_	0.85 _0.78–0.93_
*M* diff (*p*)	− 0.01 (.57)	0.01 (.82)	− 0.05 (.14)	− 0.06 (.09)
ApoA (g/L)				
BN	1.43 _1.38–1.47_	1.39 _1.34–1.44_	1.42 _1.36–1.48_	1.37 _1.29–1.44_
BED	1.41 _1.33–1.48_	1.35 _1.28–1.42_	1.42 _1.34–1.51_	1.46 _1.32–1.61_
*M* diff (*p*)	0.02 (.55)	0.04 (.19)	0 (.91)	− 0.09 (.12)
Total cholesterol/HDL				
BN	2.93 _2.85–3.01_	3.00 _2.87–3.12_	3.02 _2.88–3.16_	3.07 _2.96–3.19_
BED	3.05 _2.90–3.20_	3.06 _2.90–3.22_	3.19 _2.99–3.39_	3.19 _2.92–3.46_
*M* diff (*p*)	− 0.12 (.06)	− 0.06 (.40)	− 0.17 (.07)	− 0.12 (0.29)
ApoB/ApoA				
BN	0.57 _0.55–0.59_	0.57 _0.54–0.60_	0.58 _0.55–0.61_	0.58 _0.55–0.61_
BED	0.59 _0.56–0.62_	0.58 _0.55–0.61_	0.61 _0.57–0.66_	0.60 _0.53–0.67_
*M* diff (*p*)	− 0.02 (.16)	− 0.01 (.54)	− 0.03 (.09)	− 0.02 (.47)
Glucose (mmol/L)				
BN	4.53 _4.42–4.65_	4.59 _4.47–4.7_	4.63 _4.49–4.77_	4.48 _4.23–4.73_
BED	4.62 _4.44–4.81_	4.52 _4.33–4.7_	4.67 _4.51–4.82_	4.67 _4.49–4.85_
*M* diff (*p*)	− 0.09 (.28)	0.07 (.39)	− 0.04 (.68)	− 0.19 (0.11)
TSH (mU/L)				
BN	2.23 _1.97–2.49_	2.11 _1.90–2.33_	2.06 _1.78–2.33_	2.03 _1.70–2.36_
BED	2.51 _2.06–2.97_	2.18 _1.84–2.53_	2.21 _1.67–2.76_	2.88 _2.23–3.54_
*M* diff (*p*)	− 0.28 (.16)	− 0.07 (0.66)	− 0.15 (0.50)	**− 0.85 (.003)**
T4 (pmol/L)				
BN	14.1 _13.7–14.5_	14.1 _13.7–14.6_	14.5 _13.9–15.0_	14.5 _13.8–15.1_
BED	14.9 _14.2–15.7_	14.9 _14.0–15.8_	16.2 _14.3–18.1_	15.2 _14.5–15.8_
*M* diff (*p*)	**− 0.80 (.01)**	− 0.8 (.04)	− 1.7 (.02)	− 0.7 (.04)
Anti-TPO^1^ (kU/L)				
BN	53.5 _46.7–60.2_	60.9 _33.5–87.9_	59.3 _43.2–75.3_	72.8 _34.4–111.2_
BED	55.7 _46.8–64.5_	49.3 _43.8–55.3_	42.9 _15.5–70.3_	47.3 _38.0–56.6_
*M* diff (*p*)	− 2.2 (.68)	11.6 (.32)	16.4 (.17)	25.5 (.10)

*M*_*diff*_ mean difference between BN and BED subjects, *p* probability value. *na* not estimable as the significant and included *remission* covariate lacks data for this time-point. Cholesterol-total level, *HDL* high density lipoprotein, *LDL* low density lipoprotein, *ApoB* apolipoprotein B and *ApoA* apolipoprotein A, TSH-thyroid stimulating hormone, T4-thyroxine, *Anti-TPO* anti-thyroid peroxidase (^1^Nine cases with extreme scores (= 1300) were removed), bold numbers indicate significant differences between diagnoses

### Differences between treatments and change across time

Neither the *Group* nor the Group*Time effects were significant in any tests indicating no intervention effects with regard to lipid or metabolic outcome. The *Time* factor was significant in some base models, but were in general non-significant after adding covariates (Table 3). The most important covariate was the baseline score, thus implying a rather flat development post-treatment to follow-up with regard to treatment-related effects (see Table 4). A significant reduction in lipid levels from baseline to post-treatment (17 weeks) was however observed for TC (*M*_diff_ = − 0.23, *p* < 0.001), LDL (*M*_diff_ = -0.17, *p* = 0.002), HDL (*M*_diff_ = − 0.11, *p* < 0.001), Apo-B (*M*_diff_ = − 0.03, *p* = 0.006) and Apo-A (*M*_diff_ = -0.06, *p* = 0.003), but these changes were temporary and returned to baseline levels at later follow-ups. The only exception was the TC/HDL-ratio which steadily increased until the last follow-up (*M*_diff_ = 0.14, *p* < 0.001).

**Table 3:** Statistical significance of the study effects with regard to lipoprotein and metabolic outcomes

	Total cholesterol (mmol/L)		LDL (mmol/L)		HDL (mmol/L)		Triglycerides (mmol/L)		ApoB (g/L)		ApoA (g/L)	
Design effects (*p*-values)	Crude^a^	Adj	Crude^a^	Adj	Crude^a^	Adj	Crude^a^	Adj	Crude^a^	Adj	Crude^a^	Adj
Grp	.12	.53	.018	.55	.27	.58	.02	.20	.02	.24	.45	.69
Time	**.009**	.02	**.01**	.11	.03	.83	.09	.13	**.002**	**.002**	.04	.58
Grp*Time	.43	.88	.57	.83	.80	.57	.49	.62	.44	.54	.32	.47
Covariates (beta weights)	Crude^b^		Crude^b^		Crude^b^		Crude^b^		Crude^b^		Crude^b^	
Baseline score	.73^***^	.74^***^	.75^***^	.75^***^	.73^***^	.71^***^	.61^***^	.55^***^	.73^***^	.75^***^	.62^***^	.61^***^
Diagnosis (0-BN, 1-BED)	.16	.75^**^	.26	–	− .08	–	.25^**^	–	.04	–	− .02	–
Remission (0-no, 1-yes)	− .26^**^	− .22^**^	-.29^**^	-.24^**^	− .04	.10	− .06	–	− .04	–	− .04	.00
Purging (Z-score)	− .05	–	− .04	–	− .01	-	− .03	-	− .01	–	− .01	–
VAT (kg)	.13	–	**.43 **^**2**^	–	− .27^***^	-	.50^***^	**.25 **^**3**^	.08	–	− .06	–
Interaction (*p*-values)	Crude^c^		Crude^c^		Crude^c^		Crude^c^		Crude^c^		Crude^c^	
Diagnosis*Grp		.67	.56		.02		.72		.41		.32	
Diagnosis*Time		**.01**	.14		.16		.29		.32		.14	
Diagnosis*Grp*Time		.58	.71		.86		.80		.54		.60	
Remission*Grp	.73		.61			.90	.12		.56			.25
Remission*Time	.11		.16			**.01**	.51		.05			** < .001**
Remission*Grp*Time	.85		.67			.50	.23		.53			.53

	Total cholesterol/HDL		ApoB/ApoA		Glucose (mmol/L)		TSH (mU/L)		T4 (pmol/L)		Anti-TPO (kU/L)	
Grp	**.005**	.29	**.01**	.03	.78	.93	.71	.99	.93	.57	.17	.68
Time	**.01**	.02	.55	.51	.32	.23	.17	.18	.09	.06	.55	.44
Grp*Time	.91	.99	1.00	.97	.80	.81	.94	.96	.59	.64	.57	.61
Covariates (beta weights)	Crude^b^		Crude^b^		Crude^b^		Crude^b^		Crude^b^		Crude^b^	
Baseline	.88^***^	.90^***^	.85^***^	.88^***^	.58^***^	.58^***^	.47^***^	.47^***^	.57^***^	.55^***^	.78^***^	**.78 **^**3**^
Diagnosis (0-BN, 1-BED)	.34**	–	.05	–	.28^**^	-	.43^**^	–	1.14^***^	.96^***^	6.3	–
Remission (0-no, 1-yes)	− .08	–	− .03	–	− .04	−	.10	–	− .13	–	− 1.7	–
Purging *z-*score	− .04	–	.00	–	− .04	–	− .14	–	− .31	–	− 2.1	–
VAT (kg)	.66***	–	.11	–	.39	–	.25	–	1.98^***^	–	15.9	–
Interaction (*p-*values)	Crude^c^		Crude^c^		Crude^c^		Crude^c^		Crude^c^		Crude^c^	
Diagnosis*Grp	.04		.20		.72		.96		.34		.48	
Diagnosis*Time	.78		.73		.15		.08		.56		.21	
Diagnosis*Grp*Time	.83		.91		.33		.91		.97		.32	
Remission*Grp	.66		.91		.72		.91		.38		.78	
Remission*Time	.66		.11		.36		.09		.72		.27	
Remission*Grp*Time	.59		.63		.92		.27		.24		.77	

^**^p < .01 and ^***^p < .001 (significant effects flagged as bold).—(hyphen) = non-significant covariate effects. crude ^a^ = model with *Group*, *Time* and Group*Time as fixed effects, crude ^b^ = tests of simple associations between covariates and the outcome. crude ^c^ = *p*-values of the full 3-way interaction effects. adj = final model adjusted for significant covariates and interaction effects. The covariates *Responder* and *Purging* were repeatedly measured, thus treated as time-variant, whereas *Diagnosis* and *Vat* (kg) were baseline covariates. Cholesterol-total level, *HDL* high density lipoprotein, *LDL* low density lipoprotein, *ApoB* apolipoprotein B and ApoA-apolipoprotein A, *TSH* thyroid stimulating hormone, *T4* thyroxine, *Anti-TPO* anti-thyroid peroxidase (^1^Nine cases with extreme scores (= 1300) were removed), bold numbers indicate significant findings

**Table 4:** Lipoprotein and metabolic levels across time and treatment groups (based on final adjusted model)

		Estimated marginal means _99% confidence interval_				
		Baseline	Mid 8 week	Post 17 week	26 week	52 week
Total cholesterol (mmol/L)						
PED-t	Base	4.87 _4.63–5.12_	4.74 _4.50–4.97_	4.51 _4.25–4.77_	4.78 _4.47–5.08_	4.73 _4.35–5.10_
CBT		4.49 _4.23–4.75_	4.50 _4.19–4.80_	4.43 _4.15–4.72_	4.59 _4.23–4.96_	4.41 _3.97–4.84_
PED-t	Adj	4.82	na	4.51 _4.33–4.70_	4.72 _4.49–4.95_	4.70 _4.48–4.92_
CBT				4.59 _4.28–4.89_	4.82 _4.53–5.12_	4.74 _4.38–5.09_
LDL (mmol/L)						
PED-t	Base	3.14 _2.87–3.40_	3.01 _2.76–3.25_	2.85 _2.59–3.11_	3.08 _2.77–3.38_	3.09 _2.73–3.46_
CBT		2.66 _2.41–2.90_	2.71 _2.45–2.98_	2.64 _2.39–2.88_	2.80 _2.44–3.15_	2.66 _2.32–3.01_
PED-t	Adj	3.00	na	2.77 _2.61–2.94_	2.90 _2.66–3.14_	2.89 _2.65–3.12_
CBT				2.84 _2.62–3.05_	3.01 _2.74–3.27_	2.90 _2.57–3.22_
HDL (mmol/L)						
PED-t	Base	1.62 _1.49–1.75_	1.59 _1.47–1.71_	1.50 _1.37–1.63_	1.53 _1.40–1.66_	1.50 _1.36–1.64_
CBT		1.66 _1.53–1.79_	1.63 _1.49–1.77_	1.59 _1.46–1.72_	1.63 _1.48–1.78_	1.57 _1.37–1.76_
PED-t	Adj	1.68	1.63 _1.56–1.71_	1.54 _1.46–1.62_	1.57 _1.48–1.65_	1.53 _1.41–1.65_
CBT			1.64 _1.55–1.74_	1.59 _1.51–1.67_	1.61 _1.50–1.73_	1.54 _1.39–1.70_
Triglycerides (mmol/L)						
PED-t	Base	1.10 _0.94–1.27_	1.09 _0.95–1.23_	1.05 _0.91–1.20_	1.23 _1.03–1.44_	1.14 _0.90–1.39_
CBT		0.92 _0.81–1.03_	0.96 _0.84–1.07_	0.95 _0.79–1.10_	0.99 _0.82–1.15_	0.91 _0.67–1.15_
PED-t	Adj	1.03	1.05 _0.94–1.16_	0.99 _0.88–1.10_	1.17 _1.01–1.32_	1.08 _0.83–1.34_
CBT			1.01 _0.90–1.11_	0.98 _0.85–1.11_	1.03 _0.87–1.19_	0.99 _0.79–1.19_
ApoB (g/L)						
PED-t	Base	0.85 _0.80–0.91_	0.83 _0.77–0.88_	0.79 _0.73–0.84_	0.85 _0.78–0.91_	0.84 _0.76–0.92_
CBT		0.75 _0.69–0.80_	0.76 _0.71–0.81_	0.75 _0.69–0.80_	0.80 _0.72–0.87_	0.77 _0.69–0.84_
PED-t	Adj	0.82	0.80 _0.77–0.83_	0.76 _0.72–0.80_	0.82 _0.77–0.86_	0.81 _0.76–0.86_
CBT			0.81 _0.78–0.85_	0.80 _0.75–0.84_	0.84 _0.79–0.90_	0.82 _0.75–0.88_
ApoA (g/L)						
PED-t	Base	1.42 _1.35–1.49_	1.41 _1.34–1.48_	1.34 _1.27–1.41_	1.39 _1.32–1.47_	1.39 _1.28–1.50_
CBT		1.42 _1.35–1.50_	1.40 _1.33–1.47_	1.40 _1.32–1.48_	1.45 _1.35–1.54_	1.41 _1.28–1.53_
PED-t	Adj	1.44	1.42 _1.37–1.47_	1.35 _1.29–1.41_	1.40 _1.35–1.46_	1.40 _1.29–1.50_
CBT			1.41 _1.36–1.47_	1.40 _1.34–1.47_	1.44 _1.36–1.52_	1.39 _1.29–1.49_
Total cholesterol/HDL						
PED-t	Base	3.15 _2.93–3.38_	3.11 _2.87–3.35_	3.15 _2.90–3.41_	3.25 _3.00–3.50_	3.26 _2.97–3.54_
CBT		2.80 _2.61–2.99_	2.86 _2.67–3.05_	2.90 _2.69–3.12_	2.93 _2.68–3.19_	2.94 _2.68–3.21_
PED-t	Adj	2.97	2.94 _2.84–3.05_	2.99 _2.85–3.13_	3.07 _2.91–3.22_	3.09 _2.93–3.25_
CBT			3.01 _2.90–3.11_	3.06 _2.93–3.19_	3.10 _2.93–3.28_	3.14 _2.97–3.30_
ApoB/ApoA						
PED-t	Base	0.61 _0.56–0.66_	0.60 _0.55–0.65_	0.60 _0.54–0.65_	0.61 _0.56–0.66_	0.62 _0.55–0.68_
CBT		0.53 _0.49–0.56_	0.55 _0.51–0.59_	0.54 _0.5–0.59_	0.56 _0.50–0.62_	0.56 _0.50–0.62_
PED-t	Adj	0.57	0.57 _0.54–0.59_	0.56 _0.53–0.60_	0.58 _0.54–0.61_	0.57 _0.53–0.61_
CBT			0.59 _0.56–0.61_	0.59 _0.56–0.62_	0.60 _0.56–0.65_	0.60 _0.56–0.64_

Changes in lipoprotein levels across the two intervention groups						
Glucose (mmol/L)						
PED-t	Base	4.67 _4.49–4.85_	4.60 _4.44–4.75_	4.62 _4.45–4.78_	4.64 _4.48–4.79_	4.51 _4.28–4.73_
CBT		4.60 _4.47–4.74_	4.54 _4.37–4.72_	4.53 _4.35–4.71_	4.62 _4.40–4.85_	4.49 _4.16–4.81_
PED-t	Adj	4.63	4.57 _4.46–4.69_	4.58 _4.44–4.73_	4.63 _4.51–4.75_	4.50 _4.29–4.70_
CBT			4.56 _4.40–4.72_	4.53 _4.39–4.68_	4.65 _4.47–4.83_	4.56 _4.26–4.86_
TSH (mU/L)						
PED-t	Base	2.25 _1.93–2.57_	2.36 _2.02–2.7_	2.20 _1.90–2.51_	2.09 _1.71–2.48_	2.35 _1.82–2.88_
CBT		2.02 _1.79–2.26_	2.28 _1.9–2.67_	2.05 _1.77–2.34_	2.11 _1.74–2.48_	2.31 _1.73–2.90_
PED-t	Adj	2.18	2.33 _2.04–2.61_	2.17 _1.88–2.46_	2.11 _1.74–2.47_	2.26 _1.77–2.75_
CBT			2.35 _1.99–2.71_	2.10 _1.88–2.33_	2.12 _1.76–2.48_	2.29 _1.82–2.76_
T4 (pmol/L)						
PED-t	Base	14.9 _14.3–15.4_	14.6 _14.0–15.1_	14.3 _13.7–15.0_	15.0 _13.8–16.2_	14.7 _13.9–15.5_
CBT		14.4 _13.8–14.9_	14.2 _13.5–14.9_	14.5 _13.8–15.2_	15.0 _13.9–16.0_	14.6 _13.7–15.5_
PED-t	Adj	14.7	14.6 _14.1–15.1_	14.3 _13.7–15.0_	15.2 _14.0–16.3_	14.8 _14.2–15.5_
CBT			14.5 _13.9–15.2_	14.7 _14.1–15.3_	15.3 _14.5–16.1_	14.9 _14.1–15.7_
Anti-TPO^1^ (kU/L)						
PED-t	Base	61.0 _39.4–94.3_	60.3 _39.0–93.3_	65.3 _35.8–119.1_	58.7 _39.5–87.3_	66. 7 _32.0–139.0_
CBT		43.3 _40.2–46.6_	45.0 _40.6–49.9_	43.3 _38.2–49.2_	43.6 _36.9–51.5_	49.1 _29.5–81.5_
PED-t	Adj	54.2	55.1 _46.6–63.6_	60.1 _33.0–87.1_	52.7 _30.1–75.3_	66.2 _25.0–107.5_
CBT			53.6 _47.7–59.5_	51.9 _45.3–58.6_	53.6 _45.1–62.0_	62.2 _34.9–89.4_

*Base* estimated means for base model, *adj* estimated means adjusted for baseline and significant covariates/moderation effects. *na* not estimable as responder status data were unavailable. Cholesterol-total level, *HDL* high density lipoprotein, *LDL* low density lipoprotein, *ApoB* apolipoprotein B and *ApoA* apolipoprotein A, *TSH* thyroid stimulating hormone, *T4* thyroxine, *Anti-TPO* anti-thyroid peroxidase (^1^ Nine cases with extreme scores (= 1300) were removed)

Among the other covariates, several simple associations with the outcome in question covaried significantly in the expected direction, such as ED *diagnosis* (BED subjects having a poorer lipid or metabolic profile than BN subjects), *remission* status and visceral adipose tissue (more favorable profile among responders than non-responders, and among those with less VAT than more VAT, respectively). Purging behavior was not related to any outcome data. In the final adjusted model, several of these associations were nullified after adding baseline as covariate (see Table 3 for an overview).

### Treatment moderator effects related to status of remission

Similar as for pre-treatment diagnostic status, *remission* status did not act as an effect-modifier. It did however covary to some extent with Time, as contrast analyses indicated a worsening at follow-up (26 weeks) in most lipid measures (Table 5) among non-responders but not among responders. These negative changes in the non-responder group were however short-lived as all significant differences turned non-significant at final follow-up (52 weeks).

**Table 5:** Development of lipoprotein and metabolic levels depending on remission status (based on final adjusted model)

	Estimated marginal mean _99% confidence interval_		
	Post 17 week	26 week	52 week
Total cholesterol (mmol/L)			
Non-responders	4.67 _4.48–4.85_	4.93 _4.73–5.12_	4.72 _4.40–5.04_
Responders	4.52 _4.24–4.80_	4.43 _4.12–4.74_	4.59 _4.34–4.84_
*M* diff (*p*)	0.15 (.22)	**0.50 (< .001)**	0.13 (.39)
LDL (mmol/L)			
Non-responders	2.87 _2.72–3.01_	3.12 _2.91–3.32_	3.05 _2.80–3.30_
Responders	2.75 _2.51–3.00_	2.71 _2.38–3.03_	2.70 _2.42–2.99_
*M* diff (*p*)	0.12 (.28)	**0.41 (.005)**	0.35 (.02)
HDL (mmol/L)			
Non-responders	1.59 _1.52–1.66_	1.64 _1.56–1.72_	1.54 _1.41–1.68_
Responders	1.59 _1.50–1.69_	1.50 _1.37–1.63_	1.62 _1.51–1.74_
*M* diff (*p*)	0 (.94)	**0.14 (.01)**	-0.08 (.21)
Triglycerides (mmol/L)			
Non-responders	1.01 _0.90–1.13_	1.11 _0.98–1.24_	1.00 _0.83–1.17_
Responders	0.92 _0.81–1.02_	1.07 _0.89–1.25_	1.07 _0.79–1.34_
*M* diff (*p*)	0.09 (.11)	0.04 (.63)	-0.07 (.57)
ApoB (g/L)			
Non-responders	0.79 _0.76–0.83_	0.86 _0.82–0.91_	0.84 _0.78–0.89_
Responders	0.78 _0.74–0.83_	0.77 _0.70–0.83_	0.79 _0.73–0.86_
*M* diff (*p*)	0.01 (.71)	**0.09 (.002)**	0.05 (.19)
ApoA (g/L)			
Non-responders	1.39 _1.34–1.45_	1.47 _1.41–1.52_	1.39 _1.31–1.47_
Responders	1.38 _1.32–1.45_	1.33 _1.26–1.39_	1.45 _1.34–1.55_
*M* diff (*p*)	0.01 (.80)	**0.14 (< .001)**	− 0.06 (.24)
Total/HDL			
Non-responders	3.03 _2.92–3.14_	3.09 _2.95–3.23_	3.15 _3.02–3.28_
Responders	2.95 _2.75–3.15_	3.02 _2.76–3.28_	2.99 _2.77–3.20_
*M* diff (*p*)	0.08 (.35)	0.07 (.57)	0.16 (.07)
ApoB/ApoA			
Non-responders	0.58 _0.55–0.60_	0.60 _0.56–0.63_	0.61 _0.58–0.64_
Responders	0.58 _0.54–0.62_	0.58 _0.52–0.65_	0.55 _0.49–0.61_
*M* diff (*p*)	0.00 (.99)	0.02 (.62)	0.16 (.07)
Glucose (mmol/L)			
Non-responders	4.58 _4.46–4.70_	4.65 _4.53–4.77_	4.50 _4.25–4.74_
Responders	4.47 _4.31–4.63_	4.57 _4.36–4.78_	4.57 _4.40–4.73_
*M* diff (*p*)	0.11 (.15)	0.08 (.40)	− 0.07 (.52)
TSH (mU/L)			
Non-responders	2.08 _1.83–2.32_	2.01 _1.70–2.31_	2.42 _1.93–2.91_
Responders	2.17 _1.89–2.46_	2.44 _1.92–2.96_	2.17 _1.68–2.67_
*M* diff (*p*)	− 0.09 (.49)	-0.43 (.06)	− 0.25 (.34)
T4 (pmol/L)			
Non-responders	14.5 _13.8–15.3_	15.4 _14.3–16.6_	14.8 _14.1–15.5_
Responders	14.5 _14.0–15.0_	15.2 _14.5–15.9_	15.0 _14.3–15.6_
*M* diff (*p*)	0 (.82)	0.2 (.68)	− 0.2 (.50)
Anti-TPO^1^ (kU/L)			
Non-responders	58.0 _36.6–79.4_	51.5 _44.9–58.1_	62.8 _35.5–90.2_
Responders	49.5 _40.6–58.5_	55.3 _43.7–66.9_	57.1 _46.9–67.5_
*M* diff (*p*)	8.5 (.13)	-3.8 (.33)	5.7 (.49)

*M*_*diff*_ mean difference between responders and non-responders, *p* probability value. Cholesterol-total level, *HDL* high density lipoprotein, *LDL* low density lipoprotein, *ApoB* apolipoprotein B and *ApoA* apolipoprotein A, *TSH* thyroid stimulating hormone, *T4* thyroxine, *Anti-TPO* anti-thyroid peroxidase (^1^Nine cases with extreme scores (= 1300) were removed), bold numbers indicate significant differences between responders and non-responders

## Discussion

The present study examined the plasma profile of lipids, glucose, and thyroid hormones in women with BN or BED, effect from treatment and any possible differences in effect between CBT or PED-t, as well as the impact of pre-treatment diagnosis or status of remission. Overall, the mean levels of blood lipids and thyroid levels were within a normative range; yet, at pre-treatment more than a third had lipid values above the recommended levels for TC and LDL-c, and low HDL was more frequent among women with BED compared to women with BN. Moreover, we observed significantly higher TC and TSH among participants with BED as compared to patients with BN at last follow-up. While lipids were acutely improved after a period with treatment, there were neither any sustainable change, nor any differences between PED-t and the CBT on any of the outcomes. Rather, TC/HDL ratio was increasing throughout the period with treatment and follow-up, with a significant higher ratio at last measurement. Being in *remission* from BN or BED demonstrated some improvements in blood lipid levels compared to non-remission, however with only temporary effects lost at last follow-up.

### Impact of pre-treatment diagnosis

The normal levels of cholesterol levels (TC and LDL-c) among women with BN or BED confirm previous findings [7, 9, 37, 38]. Yet, the simple group mean picture may be deceptive given the relatively high variability in these measures, as we identified a considerable proportion of women having TC and LDL-c values above the recommended levels; in line with previous findings [7, 9]. Partly supporting our first hypothesis, was a more impaired lipid profile in women with BED compared to BN (i.e., higher number with low HDL at baseline, and a more impaired average TC during follow-up). Additionally, a tendency towards higher LDL was observed at last measurement, a finding which may have been limited from low statistical power due to dropouts. Previous publication from this patient sample identified a higher level of VAT among patients with BED compared to BN [39], and while VAT did not turn out as a significant covariate in the current adjusted models, there were some outcome-associations in the crude model that aligns with acknowledged associations of VAT to lipid levels (here: LDL, HDL and TG) [40]. Within our sample, we have observed several participants with BED having high level of VAT, which does not respond to treatment (or remission). Considering the current finding on lipid levels, including tendencies in the one-year changes, and knowing of their increased risk for type 2 diabetes and metabolic syndrome [10–12], further exploration of somatic health, effective and more holistic interventions by approaching both somatic and mental health, and response to treatment, are recommended.

No indication of an impaired thyroid status partly contradicted our first hypothesis. Comparable to previous findings [41, 42], the mean level of T4 was within the normal range, as was the mean level of TSH. On the other hand, 15.4% of participants with BED had symptoms of subclinical hypothyroidism pre-treatment, which is noticeable in view of the 1% prevalence of untreated hypothyroidism and a total frequency of 5–9% in the general female population [34, 43]. High levels of anti-TPO occurred in 14 participants (9.3%) at pre-treatment, with no improvement during follow-up. A high anti-TPO indicates a previous or current autoimmune response towards the thyroid glandule, and this may prospectively compromise the regulation of thyroid metabolism and result in a hypothyroid condition. For a period, high anti-TPO may co-occur with high TSH and T4, but these may slowly be reduced as the thyroid gland perishes. Because of the slow progress in thyroid diseases, the current findings may suggest a need for longitudinal follow-up.

### Effects from type of treatment

There was an overall favorable change in lipid profiles for the total sample when comparing pre-treatment with post-treatment 17 weeks. Nevertheless, the change was short-lived, as no significant differences to pre-treatment were found for any of the follow-up measurements. Given that the group mean levels of lipids [32] and total cholesterol [45] were within the normal range in both intervention groups, any further improvements irrespective of treatment modality may be unrealistic on group level. Nevertheless, the TC/HDL ratio increased towards the last follow-up, which is unfavorable and potential detrimental by increasing the individuals’ risk of cardiovascular diseases. Furthermore, the hypothesis of a greater effect from the PED-t compared to CBT in terms of improvements in blood lipids, glucose and thyroid hormones, was not supported. One explanation for this may be the low volume of aerobic exercise, and that the differences in volume of resistance training and increased muscle strength and muscle mass between PED-t and CBT groups were insufficient to produce the expected effect on metabolic health [25, 44]. We have previously documented a low level of aerobic physical activity which is not changing by time in this group of participants [27, 39]. While results from changes in diet is not included herein, previous exploration of this within the current sample, points to few and minor changes [46], hence an unlikely effect on metabolic profile.

### Effect from treatment outcome

The small beneficial effects on metabolic profile from remission after treatment had only minor clinical importance. This stands in contrast to a previous study [37], which reported a reduction or stabilization of glucose and lipid profiles three years after BN-treatment, and with slightly more favorable outcome among those *recovering* from BN [37]. A plausible explanation for these differences is the increase in BMI and VAT by time in our sample [23], which was not reported in the former study [37].

## Strength and limitations

The strength of this study was the RCT design, the inclusion of two ED-diagnoses providing a more nuanced insight according to ED diagnosis (i.e., BN versus BED), and the one-year follow-up time after treatment. However, the fact that only 30% (PED-t) and 20% (CBT) attended at follow-up (Fig. 1) is an important limitation. The ramifications of such a loss of study participants are most probably loss of power; hence, the risk of not detecting true effects or differences is probably larger than the possibility of biased results. The many statistical analyses may raise the risk for type-1 errors despite the use of wider confidence intervals; however, considering the explorative objective of the moderator analyses, this tradeoff may be acceptable. Moreover, considering the slow progress of thyroid complications, the length of the follow-up may have been insufficient. Not including total energy intake and nutrients also limits our findings, as nutrient intake is known to affect blood-borne metabolic indices. Still, unpublished exploration of nutrition and diet points to minor changes of importance [46], as addressed in the discussion. Finally, the fact that participants were recruited by defined inclusion criteria (e.g., an upper limit of BMI of 35) [29] is important for the interpretation of the present findings.

## Implications

If replicated, the current findings carry clinical implications for screening and treatment of BN and BED. Although few of our participants were categorized as obese, and the mean levels of metabolic parameters were within normal levels, a high frequency of metabolic impairments was identified in these young females. As such, clinicians should identify and treat clinical levels of, e.g., blood lipids and blood glucose in patients with BN or BED to reduce their risk for cardiovascular diseases. Females with BED seem as a core target group as they showed a worse one-year cholesterol profile. During the short follow-up period of 1 year, the increase in mean lipid levels was of minor clinical concern. Still, considering the young age as in the current sample, improved screening procedure may offer longer-term health preventive benefits. The simple addition of ordering lipid assessment in connection with blood samples that the clinical staff already routinely orders, should interfere little with the faster pace of clinical practice. Based on the existing knowledge of increased risk for non-communicable diseases in women with BN or BED [7–13], the implementation of revised screening- and follow-up procedures regarding somatic health is recommended. This may implicate a need for multidisciplinary therapy teams that include competence in lifestyle modifications to improve blood lipid levels among those at risk. We have previously documented that supervised physical exercise during treatment of BN or BED does not infer counterproductive effects on ED treatment or of compulsiveness [27, 29]. Hence, in future research and clinical work, it appears safe to continue to explore supervised and adapted exercise during ED treatment. The potential effects of regular high-intensity aerobic exercise, as one of the successful measures for metabolic health and reduced mortality, should be explored [24–26]. The present findings point to the need to further explore the mechanisms of metabolism and ED, and the potential role of exercise genomics and epigenetics as explanations for the lack of differences in metabolic aberration and adaptation following various treatment interventions [47, 48].

## Conclusions

On a group level, women with BN or BED present healthy blood lipids, blood glucose and thyroid hormones. However, we identified a high number of individuals with metabolic impairments and increased risk factors for cardiovascular diseases. A diagnosis of BED specifically raises the risk of an impaired lipid and glucose profile. While remission in terms of less bingeing and/or purging appears as important for a beneficial effect on blood lipids irrespective of the kind of treatment approach, we found only arbitrary and short-lived favorable improvements in plasma lipid levels with remission.

## Data Availability

Data are kept within the research group but can be shared on reasonable request.
